# Effects of Visual Attributes of Flower Borders in Urban Vegetation Landscapes on Aesthetic Preference and Emotional Perception

**DOI:** 10.3390/ijerph18179318

**Published:** 2021-09-03

**Authors:** Jingwei Zhuang, Lin Qiao, Xuan Zhang, Yang Su, Yiping Xia

**Affiliations:** 1 Institute of Landscape Architecture, College of Agriculture and Biotechnology, Zhejiang University, Hangzhou 310058, China; 21916209@zju.edu.cn (J.Z.); lynnq@zju.edu.cn (L.Q.); zhang_xuan@zju.edu.cn (X.Z.); 2 Institute of Urban and Rural Planning Theories and Technologies, College of Civil Engineering and Architecture, Zhejiang University, Hangzhou 310058, China; 3 The Architectural Design & Research Institute of Zhejiang University Co., Ltd., Hangzhou 310030, China; suyang@zju.edu.cn

**Keywords:** aesthetic preference, emotional perception, flower border, urban vegetation landscape, face recognition

## Abstract

The vegetation landscape in urban green space has been shown to provide great psychological benefits to people. Flower border is a well-designed small-scale vegetation landscape with the advantages of color and vegetation richness. This study focused on the effects of the visual attributes of flower borders on the aesthetic preference and emotional perception. The face recognition measurement method was used to obtain the emotional perception and the questionnaire survey method was used to measure the aesthetic preference. The results indicated the following: (1) regarding the ‘color features’ factor, high proportions of cool color and green vegetation significantly increased aesthetic preference and emotional valence, while the proportion of warm color had a negative effect on valence; (2) the ‘visual attractiveness’ (color brightness, and visual richness) and ‘color configuration’ (number of plant patches and number of color hues) factor was positively associated with aesthetic preference and emotional valence; (3) aesthetic preference was significantly related to emotional valence; (4) males expressed higher aesthetic preference and valence for flower border images than females. The results are expected to improve the aesthetic quality of flower borders and to promote public emotional health through the effective design of urban vegetation landscapes.

## 1. Introduction

Urban green space plays an increasingly important role in promoting human well-being [1,2,3,4,5,6] and relieving stress and mental fatigue [7,8]. Well-designed urban greening environment not only improves urban aesthetic quality [9] but also promotes the physical and mental health of urban residents [10]. Plants are the key environmental variable that form a major part of the urban green space [11]. Previous studies on vegetation landscapes in urban green space have mainly addressed relatively large-scale vegetation landscape, such as urban forests [12], parks [13,14] and gardens [15]. However, small-scale urban vegetation landscapes are particularly understudied in terms of the public’s aesthetic preference and emotional perception [16,17].

In recent years, flower borders, a type of small-scale vegetation landscape, have been increasingly used in urban green space in China. Flower border is one of the application forms of flowers that combines flowers, herbaceous plants, shrubs and small trees [18]. It is a natural ribbon flower arrangement with trees, hedges, low walls or architectures as the background. Flower borders were originally developed in English gardens and were widely used in parks, botanical gardens, private gardens and other places in America and many European countries. Compared with other forms like flower bed, flowering shrubs, flower belt, etc., flower borders have greater potential for increasing plant and landscape diversity [19]. They can improve ecological benefit and maintain the stability of vegetation communities. A recent study on the public evaluation of urban flower borders revealed that flower borders with higher scores are characterized by high species diversity, rich colors and distinct landscapes [20]. However, the visual landscape characteristics of flower borders have not been systematically and quantitatively studied, and it remains uncertain how these characteristics influence public aesthetic preference or emotional perception.

Previous studies on public subjective preference for vegetation landscapes have focused on characteristics such as foliage color, vegetation height and density, naturalness, and species richness [21,22,23]. However, existing knowledge of preferred vegetation characteristics may not be applicable to predicting the preference and emotional response for flower borders because of their unique composition and characteristics. Color, as a basic aspect of human perception [24], should be the most significant visual attribute that may influence emotional perception. Being the most changeable elements of nature and giving emotional perceptions are the characteristics of colors [25]. Both foliage color and flower color play an important role in determining landscape preferences and perception [26]. For example, due to the difference in emotional associations and visual quality, green-white foliage could develop negative feelings such as sadness and ugly, while green-yellow and bright green foliage could develop positive feelings like cheerful and active [27]. In the case of flower borders, the collocation and combination of different colors may influence preferences and perceptions [28]. In addition, Barnes states that cool colors (blues, greens, and purples), whites and pastel shades create a calming experience whereas hot and vibrant colors, such as bright reds, oranges and yellow, distract the mind through stimulation and are uplifting [29]. Color features, including cool and warm colors [30], and color brightness may play important roles in the visual attributes of flower borders. Likewise, color contrast has proven to be an important attribute of perceived beauty [13,31]. Furthermore, the structural composition of flower borders, including their visual richness and the distribution of plant patches, may be important in determining preference and emotional response [7,26,32]. This study seeks to identify the aesthetic preferences and emotional perceptions for different visual attributes of flower borders.

Measuring people’s perceptions and preferences has regularly used questionnaire methodologies that can be reliable to some extent [12,33]. However, in terms of emotional perception, evaluation through self-reported scores has several apparent biases from the subjective emotion of respondents, real-time moods, problematic questions, and social role-restricted results [12]. An objective method was used to obtain the emotional perception results in this study, which were then combined with the subjective preference results. Facial expressions represent an emotional response that provides a novel way to show people’s emotional perception when viewing vegetation landscapes. According to the circumplex model of affect [34], all human reactions or affective states arise from two dimensions, valence and arousal. Valence is a measure of pleasantness (positive or negative) and arousal measures the level of activation of an emotion and indicates physical and mental alertness [35]. This model has been applied to interpret human reactions to planting environments [32]. Face reading is a novel technique that analyzes visual recordings of faces through a software algorithm that was generated by training the model using big data of intended emotion expressions [12]. The current technique can achieve a facial analysis accuracy of up to 87% of the perceived emotion [36], which provides the possibility to analyze the emotional perception of small-scale vegetation landscapes.

This study seeks to explore the public aesthetic preference for and emotional perception of flower borders in the context of the urban environment in China. We selected typical flower border images to explore the impacts of visual attributes on preference and perception. The questions to be answered include the following: (1) what is the relationship among the visual attributes of flower borders in urban green space; (2) what are the effects of the visual attributes of flower borders on people’s aesthetic preference and emotional perception; (3) what is the relationship between aesthetic preference and emotional perception; (4) what effect does the gender of participants have on aesthetic preference or emotional perception? The findings could contribute to a better design of flower borders for urban greening and provide an objective evaluation method for public emotional perception of vegetation landscapes.

## 2. Materials and Methods

### 2.1. Stimuli

**Figure 1:**
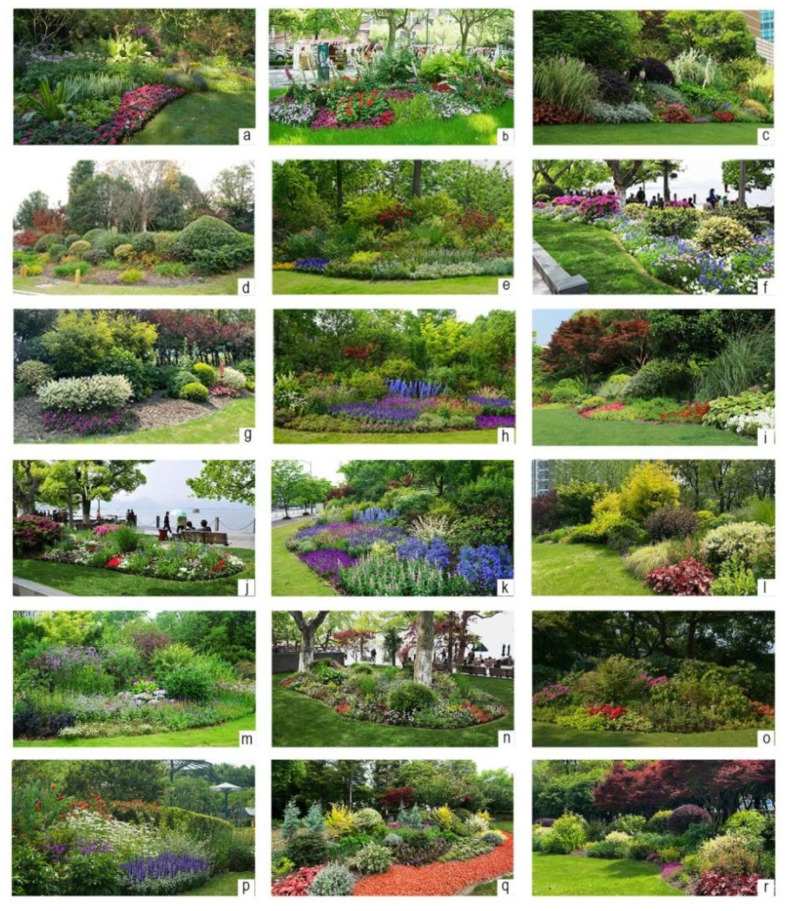
Representative scenes of flower borders used as visual stimuli in this study; (**a**–**r**) are the 18 images for this study.

Photographic images were used as surrogates for real flower border landscapes in this study as the effectiveness of this method has been widely confirmed [31,37,38]. A total of 70 images of flower borders in urban green space were taken by one of the authors using a Sony Alpha 7R II digital camera between the hours of 10.00 a.m. and 2.30 p.m. on clear days. All images were transformed to a 3936 × 2214 px resolution using the Adobe Photoshop CS6 software. The photographs were not further digitally modified. To fulfill the objectives of this study, from all photographs, 18 final images that are the most representative scenes of flower borders were selected for evaluation according to the opinions of experts on landscape architecture (Figure 1) [37].

According to the psychophysical approach, landscape is a physical–visual experience [22]. Quantifying the visual attributes of a landscape will improve the effectiveness of landscape evaluation, thus establishing the aesthetic preference and emotional perception for the landscape as a whole. Considering the characteristics of the samples, research of the literature [7,13,29,32], and advice from experts on vegetation landscapes, nine visual attributes of vegetation landscapes were finally selected, as shown in Table 1. We quantitatively evaluated the intensity of the visual attributes present in each image using categorical or continuous variables. Three categorical variables were assessed by the participants according to their perceptions using the questionnaire method (Table A1). Six continuous variables related to plant color were measured using the ColorImpact 4 software based on the HSV color model [39], which is adopted according to the human visual perception characteristics of color. We divided color hues into six categories according to the HSV color model, including red, yellow, green, cyan, blue and magenta.

### 2.2. Participants

A total of 113 participants (42 males and 71 females) aged between 18 and 52, were recruited from the Zhejiang University intramural forum for the internet surveys on aesthetic preference and 35 participants (13 males and 22 females) of them simultaneously participated in the face recognition experiment on emotional perception. The participants include college students, teachers and other school staff. The participants were carefully selected and did not have any mental illnesses, physical injuries, blindness, eye disease, color blindness or weakness.

### 2.3. Procedure

The face recognition experiment took place in a quiet room at Zhejiang University. The room was set up specifically for the purposes of this study. Prior to the study, all participants were informed of the entire experimental procedure and the relevant equipment’s function within the experiment. All participants were volunteers and asked to read and sign a consent form if they wished to participate. At the beginning of the experiment, five ‘warm-up’ images were shown first as a preview sample of the study content. A series of pilot tests preceded the main study to define and fine-tune the experimental setup.

Participants were seated in a comfortable stable chair in front of a desk approximately 60 cm away from a 32″ full HD monitor with an adjustable height. Visual stimuli of 18 images were displayed at random. Each trial began with the display of a white image for 10 s, used as a baseline, followed by the display of the stimuli for 8 s; this process was repeated throughout the trial. Finally, the whole trial ended with an additional 10 s black image. A digital camera (Sony Alpha 7R II) was positioned to record the facial expressions of the participants above and behind the monitor.

After the face recognition experiment, each participant received an online questionnaire survey. The participants were asked to rate their aesthetic preferences for each image. They were instructed to rate the color brightness, color contrast and visual richness of the 18 images according to the measurement scale shown in Table 1. There was no time limit to rate each image. When the ratings were finished, the demographic information of participants was requested. After the internet survey, each participant received 10 RMB as a reward.

### 2.4. Aesthetic Preference and Emotional Perception Measurement

In this study, the participants’ aesthetic preferences for each image of flower borders were collected through a questionnaire survey. This method has been widely used by previous researchers, and its reliability has been generally accepted [40,41]. We measured the aesthetic preference for images of flower border as the degree to which participants agreed with the statement ‘this vegetation landscape is very beautiful’. Participants were asked to rate the 18 images on a 5-point rating scale ranging from 5 = strongly agree to 1 = strongly disagree (Table A1). The ratings were used as the dependent variable representing aesthetic preference in the post hoc analysis.

Facial expression measurements were used to generate the valence and arousal of the participants when viewing the 18 images of flower borders. The valence measures the level of pleasure while the arousal indicates the level of affective activation [42]. Based on the large-scale Aff-Wild2 dataset and the Affective Behavior Analysis in-the-wild, we used the stat and temporal module to fine-tune face features again to establish the emotion expression analysis model [43]. More details about the mechanism and training methods of the model can be found in Do Nhu and Kim [43]. This measure analyzes the videos of participants’ facial reactions during the experiment frame-by-frame and predicts the emotion with small displacements in the faces. The model eventually outputs the valence and arousal of each participant for each frame. We calculated the average valence and arousal of each flower border image and white image to obtain the emotional fluctuation of the participants when viewing the flower border landscapes. The values of the changes in valence and arousal were used as two dependent variables related to emotional perception in the post hoc analysis.

### 2.5. Statistical Analysis

All statistical analyses were performed using SPSS 20 software. The mean value for each visual attribute in each image was calculated and recorded. To abstract the main characteristics of the urban flower border landscapes, principal component analysis (PCA) was performed using the normalized varimax rotation procedure. This method allows redundancies among visual attributes to be detected and reveals the potential dimensions of the data. One-way analysis of variance (ANOVA) and correlation analysis were employed to examine the differences among emotional perception, including valence and arousal, and aesthetic preference with various visual attributes of the flower borders. We performed Levene’s test of equality of variances to quantify the homogeneity of the variance. A general linear model was used to explore the significant predictors of aesthetic preference and emotional perception. The final minimum adequate models were obtained via the backward elimination of nonsignificant (*p* > 0.05) variables. Correlation analysis was then used to identify associations between aesthetic preference and valence, arousal and aesthetic preference, and valence and arousal. In addition, an independent-sample t-test was used to examine the differences in aesthetic preference, valence and arousal in different gender groups of respondents.

## 3. Results

### 3.1. Relationships among Visual Attributes

The main factors of flower borders were identified by applying the statistical PCA procedure to the visual-attribute ratings of the 18 images. Factor scores were generated using a varimax rotation. The result of Bartlett’s test (Bartlett = 12,250.21, *p* < 0.0001) proved that the variables can be analyzed using a factor analysis model. The Kaiser–Meyer–Olkin (KMO = 0.685 > 0.5) value demonstrated that the factor analysis results were reliable. This process determined that the three factors that accounted for approximately 79.42% of the total data variance were extracted. Table 2 illustrates the factor loadings and communalities for the items. The factor loadings range from 0.63 to 0.97 and the communalities varied from 0.63 to 0.94, which shows that some visual attributes reflect the same landscape features.

The first factor, explaining 30.63% of the variance, could be interpreted as the ‘color features’, which are related to the proportion of cool colors, warm colors and green vegetation. The second factor, explaining 26.68% of the variance, could be interpreted as ‘visual attractiveness’. This factor embodies the following three variables: visual richness, color brightness and color contrast. The third factor, explaining 22.11% of the variance, could be explained as ‘color configuration’, which is related to the number of colors, color hues and plant patches. The factor analysis revealed the four main landscape visual characteristic factors of flower borders.

### 3.2. Effects of Visual Attributes on Aesthetic Preference and Emotional Perception

The one-way ANOVA showed that aesthetic preference differed significantly across the images due to differences in the ‘visual attractiveness’ factor. The images with higher color brightness scores were rated significantly higher than the images with lower brightness, and the images with higher color contrast were ranked significantly higher than those with lower contrast. Additionally, visual richness significantly increased aesthetic preference (Table 3). Regarding emotional perception, the ‘visual attractiveness’ factor had a significant effect on valence but no significant effect on arousal. The images with color brightness rated 5 elicited significantly higher emotional valence than the images with color brightness rated 2 and 3. Additionally, the images with visual richness of 5 points experienced significantly higher valence than those with visual richness of 2 points. Color contrast had no significant effect on valence (Table 3). The result of observed power (observed power = 1 for aesthetic preference and observed power = 0.66 of CB for valence, observed power = 0.77 of VR for valence) proved that our sample was able to make a good judgment of the statistical results [44].

The correlation analysis of the effects of the ‘color features’ and ‘color configuration’ factors, including six visual attributes, on aesthetic preference and emotional perception is presented in Table 4. According to the results, the proportion of green vegetation, the proportion of cool colors, the number of color hues and the number of plant patches have positive effects on the aesthetic preference and valence. The number of colors has a positive effect on aesthetic preference but has no significant effect on valence while the proportion of warm colors only has a negative effect on valence. The correlation analysis shows that none of these factors significantly influenced arousal (Table 4).

A backward multiple regression analysis using all the related visual attributes as independent variables and aesthetic preference as the dependent variable was conducted. As shown in Table 5, ‘visual richness’, ‘color brightness’, ‘color contrast’, ‘proportion of green vegetation’, ‘proportion of cool color’, ‘number of plant patches’ and ‘number of color hues’ were significant predictors of aesthetic preference accounting for 44% of the variance (F = 224.397, *p* < 0.001). This result suggested that three factors ‘visual attractiveness’, ‘color features’ and ‘color configuration’ interactively influence aesthetic preference. By referring to values in Arriaza et al. [30] (value of tolerance < 0.2 or VIF > 10, which indicates a problem), there was no multicollinearity problem in our models (the minimal value of tolerance = 0.230 > 0.2 and the maximal VIF = 4.352 < 10). Thus, our models were acceptable. Then, two regression analyses were performed with valence and arousal of emotional perception as the dependent variable and all the visual attributes of flower borders as independent variables. The R-squared was low (R2 ≈ 0.1), suggesting that apart from the three landscape characteristic factors, other predictors that were not included in this study affect emotional perception.

### 3.3. Relationship between Aesthetic Preference and Emotional Perception

**Figure 2:**
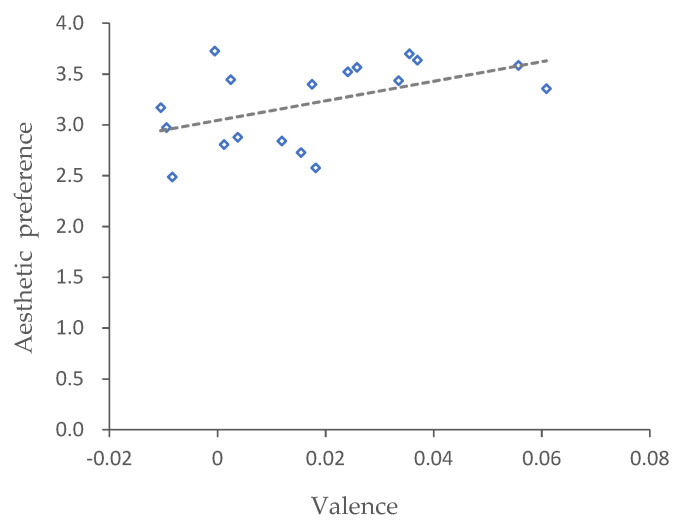
Correlation between aesthetic preference and emotional perception.

The correlation analysis indicated that there was a significantly positive correlation between aesthetic preference and valence (R = 0.51, *p* < 0.005) (Figure 2). This meant that for the flower borders in urban green spaces, the aesthetic preference value increased with the valence value related to emotional perception and vice versa. However, there were no significant correlations between arousal and aesthetic preference (*p* > 0.05) or valence (*p* > 0.05).

### 3.4. Differences in Aesthetic Preference and Emotional Perception Based on Gender

A t-test was performed to check for any differences in aesthetic preference and emotional perception based on gender. The results showed that the observed power = 0.842 (>0.8) for aesthetic preference and the observed power = 0.99 (>0.8) for valence. The results suggested that gender had no significant effect on arousal but had a significant effect on aesthetic preference and valence. Males gave significantly higher scores for aesthetic preference on average than females (Table 6). For emotional perception, compared with females, images of flower borders elicited higher valence among males (Table 6).

## 4. Discussion

### 4.1. Relationship between Visual Factors and Aesthetic Preference and Emotional Perception

Our research, using factor analysis, identified three main factors affecting the preference and perception of flower borders which were ‘color features’, ‘visual attractiveness’ and ‘color configuration’. Vegetation color associated with two factors, ‘color features’ and ‘color configuration’ can be seen as a key variable in flower borders. Among the three factors, ‘color features’ showed the highest factor loading, which indicated the significance of color design for flower borders. Color plays an important role in determining landscape preference [23,26], and it is a critical tool in conditioning, directing, and inducing specific psychological states to individuals [45]. For small-scale plant landscapes, flower borders are one of the few components with rich and varied color configurations and combinations. The results showed that a higher proportion of green vegetation for flower borders is preferred by people and can give people a higher level of emotional pleasure. Previous research suggested that green vegetation was highly preferred, and perceived as beautiful [46] and indicative of landscape health [26]. Furthermore, the results demonstrate that the use of cool color plants can improve people’s pleasure and preference in contrast to warm colors. This agrees with the findings that some cool colors can create a sense of vivacity while some dark warm colors can cause a sense of fear and sorrow [47]. However, the results of color temperature were also contrary to some previous studies which might be due to the visual attributes we chose [16]. Most of the previous studies selected foliage or flowers of pure cool color or warm color for comparative analysis, while our study selected the proportion of cool color or warm color as the variables of analysis. There is a clear distinction, which means designers of flower borders ought to control the proportion of cool color and warm color plants appropriately in the configuration process, rather than use cool color or warm color plants alone. According to our results, increasing the use of cool colors such as blue and purple flowers and green plants in the design of flower borders can have a beneficial effect on public emotional health.

In terms of ‘color configuration’ factor, we also find that to create flower borders with high aesthetic and emotional value in urban green space, trees, shrubs and flowers with more color hues should be selected. However, the specific number of colors has no effect on emotional perception, which means that the configuration of the main color hues should be considered more in the color design of flower borders. In addition, compared with large plant patches, the interactive planting of large numbers of small plant patches is a better choice for flower border planting design. A study of meadow preferences in Switzerland indicates that people like diverse meadows consisting of a green matrix with some colorful flowers [21], which is similar to our results to some degree. Such information would facilitate the design of flower borders in urban green space with different characteristics of landscape sites and landscape functions to satisfy the aesthetic and emotional needs of the public.

As mentioned above, the significance of color design was also reflected in the ‘visual attractiveness’ factor. Color brightness and visual richness can significantly increase people’s preference and pleasure. A previous study also revealed that bright, vivid plants were associated with a positive activated state [32]. Valence is proven to be strongly connected with brightness. The results also suggest that abundant flowers and herbaceous plants are important elements of the flower borders that promote both preference and valence which support the view that increasing diversity was associated with higher preferences. Color contrast is shown to have a positive effect on aesthetic preferences but no effect on emotional perception. High-contrast colors are considered to create a sense of vivacity and should be taken into account when choosing plant colors. The findings have practical significance for flower border design in that increasing the visual highlights can enhance the aesthetic value and emotional benefit of the public.

The regression analysis suggests that the ‘visual richness’, ‘color brightness’, ‘color contrast’, ‘proportion of green vegetation’, ‘proportion of cool colors’ and ‘number of plant patches’ are positive predictors of the aesthetic preference of flower borders while the ‘number of color hues’ is a negative predictor. This partially agrees with the ANOVA above. Nevertheless, the nine visual attributes of flower borders cannot predict the two variables of emotional perception well. This means that factors that are not considered in this study, such as the participants’ mood or their familiarity with flower borders, may have an effect on valence or arousal. Previous research also indicated that contextual factors such as the meanings people associate with places could influence their perceptions of scenes.

### 4.2. The Relationship between Aesthetic Preference and Emotional Perception

In this study, aesthetic preference can be considered as the degree of beauty and affective effect of the flower border landscape. Emotional perception refers to the identification of facial cues to the emotions of the participants when viewing the images of flower borders. For the sub-variables of emotional perception, valence in our study refers to the fluctuation value of valence, which is related to the degree of pleasure. Valence is positively related to aesthetic preference while arousal has no significant relationship with aesthetic preference. This means that flower borders with a higher degree of beauty can make people more pleasant, and vice versa. Some studies have confirmed the positive correlation between aesthetic preference and restorative potential [10,46,48], and restorative effects of landscapes involve positive changes in emotional states [15]. Thus, the improvement of the valence of emotional perception in this study can represent emotional recovery to some extent. In other words, the higher the beauty of the flower borders, the greater the potential for emotional recovery that can increase public emotional health.

Our findings provide a new way of judging public preferences and perception for urban vegetation landscapes. Previous studies on aesthetic preference and emotional perception mostly adopted subjective evaluation, which was based on the results of questionnaire methodologies. With the development of face-reading techniques, if people’s facial expressions while viewing vegetation landscapes could be monitored extensively, credible public landscape preferences based on big data can be easily obtained. Face-reading techniques can be a timely and convenient method for the government or landscape designers to obtain feedback on urban greening design and understand the aesthetic and emotional needs of the public.

### 4.3. Influence of Gender on Aesthetic Preference and Emotional Perception

Gender has a significant impact on participants’ aesthetic preferences and emotional perception with respect to flower borders. Our results demonstrate that men prefer flower borders more than women and men perceived the images being viewed as more pleasant than women. This is in contrast to previous research on flowers or colorful meadows which found that women rated significantly higher preference scores than men [49]. The reasons may be that the spatial arrangement of flower borders is usually half-closed in urban roadside space or woodland edges with a background with a high density of trees that influences the sense of safety [50] and thus affects female emotional perceptions. This is consistent with a study on woodland spaces and edges, which showed that the men gave all pictures higher ratings on perception of safety and preference than women [14].

### 4.4. Limitations and Further Research

This study focuses on investigating the relationship between visual attributes and people’s aesthetic preference or emotional perception for flower borders. The selection of plant species, the topography and the facade design are important parts of flower border design that may influence aesthetic preference or emotional perception. The use of photographs as landscape surrogates cannot provide a complete view of the vegetation landscape [40] and surrounding urban environment or reflect the physical accessibility of flower borders which are usually considered to be important predictor variables in preference studies [38]. Additionally, previous studies have shown that auditory, tactile and olfactory stimulation can also affect aesthetic quality and affective reactions [51]. These factors should be considered in future studies. Furthermore, the measurement of emotional perception used in the present study is based on a novel technique used to recognize the facial emotions of the participants. The accuracy of this technique applied in studies on vegetation landscape has not been well demonstrated to date. Additional studies developing big data of the intended emotional expressions of different population demographics in response to vegetation landscape in urban green space are needed to improve the model’s precision to obtain more accurate emotional data.

## 5. Conclusions

Due to the mental benefits of vegetation landscapes in urban green space to public health, this study focused on small-scale vegetation landscapes that have not been thoroughly studied. We employed a novel approach using facial expression analysis and a traditional questionnaire survey to investigate the effects of the visual attributes of flower borders on public aesthetic preference and emotional perception. Our results indicate that high levels of color contrast, brightness and visual richness of flower borders are associated with optimal aesthetic preference and emotional perception. High proportions of green vegetation and large amounts of color vegetation selection are also desirable, but it needs to be noted that overly dense trees in the background may lead to the perception of insecurity. Additionally, different proportions of warm or cool colors can be matched according to different site requirements. Despite some limitations, the information reported in the present study could be useful for a better design of flower borders to create a better urban environment and satisfy the needs of urban residents. Understanding public aesthetic preferences and emotional perception related to the visual attributes of flower borders can improve the design of flower borders in urban green space in view of their aesthetic and ecological advantages to optimize the urban environment.

## Figures and Tables

**Table 1:** Visual attributes of flower border including three categorical variables and six continuous variables.

Attributes	Abbreviation	Scoring				
Categorical variables		1	2	3	4	5
Color contrast	CC	Very weak	Weak	Clear	Strong	Very strong
Color brightness	CB	A Little	Little	Middle	Much	Very much
Visual richness	VR	A Little	Little	Middle	Much	Very much
Continuous variables						
Number of colors	NC	Value range 1–20				
Number of color hues	NCH	Value range 1–6				
Number of plant patches	NPP	Value range 1–50				
Proportion of green vegetation	PGV	Value range 0–100%				
Proportion of cool colors	PCC	Value range 0–100%				
Proportion of warm colors	PWC	Value range 0–100%				

**Table 2:** Principal component factor analysis of the visual attributes of flower borders in urban green space.

Items ^b^	Factor Loadings			Communality	Mean Score
	1	2	3		
VR	0.14	0.88 ^a^	0.13	0.80	3.35 ± 1.12
CB	0.03	0.90 ^a^	0.03	0.80	3.35 ± 1.08
CC	−0.02	0.89 ^a^	0.00	0.80	3.30 ± 1.09
PGV	0.82 ^a^	0.14	0.41	0.84	38.43 ± 15.37
PCC	0.97 ^a^	−0.01	0.08	0.94	22.66 ± 14.07
PWC	−0.79 ^a^	−0.02	0.08	0.63	39.71 ± 9.75
NPP	−0.16	0.14	0.82 ^a^	0.71	16.28 ± 8.76
NC	0.34	−0.02	0.82 ^a^	0.79	6.28 ± 2.35
NCH	0.63	0.05	0.67 ^a^	0.85	4.33 ± 0.88
% of variance	30.63	26.68	22.11	79.42	

^a^ Indicates highest factor loading for a variable. ^b^ Same as Table 1.

**Table 3:** Effects of ‘visual attractiveness’ factor on aesthetic preference and emotional perception.

CB ^a^	Preference	Valence	CC ^a^	Preference	Valence	VR ^a^	Preference	Valence
1	1.65 e	−0.013 ab	1	1.70 e	0.056	1	1.82 e	0.023 ab
2	2.57 d	0.004 b	2	2.61 d	−0.001	2	2.54 d	−0.002 b
3	3.07 c	0.003 b	3	3.10 c	0.018	3	3.04 c	0.009 ab
4	3.50 b	0.026 ab	4	3.56 b	0.012	4	3.53 b	0.019 ab
5	4.12 a	0.049 a	5	4.08 a	0.045	5	4.10 a	0.054 a

Significant difference at the 0.05 level is shown by different letters (a, b, c, d and e). ^a^ Same as Table 1.

**Table 4:** Effects of the ‘color features’ and ‘color configuration’ factors on aesthetic preference and emotional perception.

Visual Attribute ^a^		Preference	Valence	Arousal	Visual Attribute ^a^		Preference	Valence	Arousal
Color features					Color configuration				
PGV	Coefficients	0.82 **	0.10 *	−0.01	NC	Coefficients	0.05 **	0.05	0.01
	Significance	0.00	0.02	0.96		Significance	0.00	0.21	0.94
PCC	Coefficients	0.51 *	0.10 *	−0.01	NPP	Coefficients	0.13 **	0.10 *	−0.01
	Significance	0.02	0.02	0.86		Significance	0.00	0.02	0.77
PWC	Coefficients	−0.20	−0.09 *	0.02	NCH	Coefficients	0.07 **	0.10 *	−0.01
	Significance	0.35	0.02	0.67		Significance	0.00	0.01	0.74

*, ** Significant at *p* = 0.05 or 0.01, respectively. ^a^ Same as Table 1.

**Table 5:** Significant predictors for the aesthetic preference emerging from backward multiple linear regression analysis.

Dependent	Independent ^a^	Unstandardized Beta	Standardized Beta	t	Sig.	Collinearity Statistics	
						Tolerance	VIF
Aesthetic preference (adjusted R^2^ = 0.437)	(Constant)	1.122		10.630	0.000		
	VR	0.256	0.287	11.186	0.000	0.423	2.366
	CB	0.185	0.200	7.743	0.000	0.418	2.394
	CC	0.222	0.243	9.598	0.000	0.432	2.312
	PGV	0.004	0.069	1.970	0.049	0.230	4.352
	PCC	0.004	0.059	1.726	0.085	0.238	4.195
	NPP	0.007	0.059	2.689	0.007	0.585	1.709
	NCH	−0.113	−0.100	−3.444	0.001	0.330	3.034

^a^ Same as Table 1.

**Table 6:** Effects of the gender on aesthetic preference and emotional perception.

		Aesthetic Preference	Emotional Perception	
			Valence	Arousal
Gender				
	Male	3.31 **	0.14 **	0.25
	Female	3.17 **	0.09 **	0.25
F-value		0.11	11.81	0.46
Significance		0.004	0.000	0.919

** Significant at *p* = 0.01, respectively.

## Data Availability

The data are not publicly available due to the ongoing research, and the authors will continue to work with it in the future.

## Appendix A

**Table A1:** Questionnaire 1. Aesthetic preference and categorical visual attributes scale for the flower borders (Imagine you are in the scene presented by the image, how would you agree with the description; please tick the images with ‘√’ in your selected items).

Variable	Description	Scoring				
		1	2	3	4	5
Aesthetic preference	This vegetation landscape is very beautiful.					
Color contrast	The color contrast of this flower border is strong.					
Color brightness	The color brightness of this flower border is strong.					
Visual richness	The visual richness of this flower border is high.					
